# The Calcivis story - enamel caries activity assessment from technology to practice

**DOI:** 10.1038/s41415-021-3755-8

**Published:** 2021-12-17

**Authors:** Nigel B. Pitts, Chris Longbottom, Adam Christie, Bruce Vernon, Graham Bailey

**Affiliations:** 41415112903001 Faculty of Dentistry, Oral and Craniofacial Sciences, King´s College London, Tower Wing, Guy´s Hospital, London, SE1 9RT, UK; 41415112903002 Calcivis Ltd, Nine Edinburgh BioQuarter, 9 Little France Road, Edinburgh, EH16 4UX, UK

## Abstract

The Calcivis story is one of innovation and collaboration to deliver new technology capable of helping dentists improve patient care through solving an unmet clinical need in assessing the activity of caries lesions in enamel. Presently, there is no system routinely used in dental practice that can, in a single visit, determine whether a non-cavitated caries lesion is active or not. Calcivis has evolved since 2005, when a potential link between basic science in luminescence and differentiating initial-stage caries lesions that are actively demineralising and likely to progress, from other lesions which are inactive and currently do not need interventive care, was recognised. The 16-year journey has involved clinical academic dentists, scientists and entrepreneurs, general practitioners and their patients, together with serial investors and a core team working to patent, refine, assess and develop products to submit to regulatory approval and take to the international dental market. This journey has been made possible through effective long-term collaborations between disparate groups all sharing a common vision for the possibilities of harnessing new technology to help dental professionals provide better care for their patients.

## Key points

- Information about caries activity in non-cavitated lesions is important to help the dental team assess the care needs of both individual tooth surfaces as well as the patient as a whole.
- Assessing caries activity or inactivity is different from merely detecting the presence or absence of lesions and staging their severity; visual activity assessments of initial-stage lesions are subjective and often unreliable.
- The Calcivis-developed technology can, for the first time, provide an objective indication of enamel lesion activity related to the release of free calcium from demineralising lesions and a demineralisation map image can communicate the findings to both dentists and patients.

## Introduction

Caries remains a common chronic disease in children and older people.^1^^,^^2^ Caries diagnosis involves not only detection of lesion extent but also assessment of its activity. Currently, the latter necessitates monitoring of behaviour over time.^3^^,^^4^

Caries progression, when demineralisation (indicated by loss of calcium) outstrips remineralisation, is a dynamic process that in early stages is reversible.^1^^,^^5^^,^^6^ However, presently, there is no system routinely used in dental practice that can, in a single visit, determine whether a non-cavitated lesion is active or not, or whether there are potential areas where a lesion may be actively developing but is, as yet, undetectable with visual/tactile/radiographic assessments.^4^

The historical context should be mentioned here as the timeline of understanding dental caries (from the 1500s to today's science) is frequently not appreciated.^7^ The timeline in explaining the disease phenomenon ranges from Hippocrates in 460-377 BC via many great scientific observers including G. V. Black, Massler (understanding dentine caries), Koulourides, Marsh, Kidd and Reynolds (highlighting the remineralisation of enamel lesions).^7^

Recently, lesion activity assessment is aided by using 'scoring' systems such as the International Caries Detection and Assessment System (ICDAS) and the Nyvad criteria;^1^^,^^8^ however, both specificity and sensitivity of such systems is variable^9^ and the approach can differ between countries, depending on the grading system used and the individual dentist.^6^^,^^8^^,^^10^^,^^11^^,^^12^^,^^13^

With the advent of detailed detection and assessment techniques and better-quality digital radiographs, there is a parallel acceleration of a long-standing move (at varying rates) from restorative treatment to preventive management.^5^^,^^14^ However, carious lesions seen at an early 'diagnostic threshold,' such as white spot lesions, can still present a management dilemma.^1^^,^^8^ Immediate operative treatment brings the potential of unnecessary tooth drilling if the lesion was actually arrested and would not have progressed, but a 'watch and wait' approach involves longitudinal monitoring to see if more tooth destruction occurs, potentially leading to a more significant problem.^15^

## Carious lesion activity assessment: addressing an unmet need

The key to moving from interventional to preventive management is the ability to not only detect subclinical, subsurface demineralisation, along with more obvious lesions on intact enamel surfaces, but also to assess the activity level of such lesions.^5^ Use of a system that can reliably identify and record lesion activity fits into the current need for more evidence-based caries care.^5^^,^^16^ Radiographs can be used to detect radiolucency as a surrogate measure of lesion demineralisation and fibre-optic transillumination, whereby a bright light applied to the side of the tooth can show carious tissue as a dark shadow. However, with these tools, sequential observations at different time points (generally several months) are needed to assess lesion activity.^15^^,^^17^^,^^18^ Fluorescent indicators and illumination can identify bacterial porphyrins via a pen-like device. Similarly, as bacterial lactic acid is a principal factor in the caries process, use of impression materials with a lactic acid-sensitive colour-changing indicator can show areas of high acid productivity.^6^ However, these are only surrogate markers of lesion activity, since they indicate potentially cariogenic bacterial activity, not demineralisation *per se.*

### Bioluminescent photoproteins to detect lesion activity in enamel

In 2005, the unmet need to assess the carious lesion activity of non-cavitated lesions in a single dental examination was recognised by Professor Pitts and Dr Longbottom (then Dundee academics). Following the chance discovery of the book *Glowing genes*,^19^ the idea of bioluminescent photoproteins that reacted to the presence of free calcium ions (Ca2^+^) was uncovered. Technology using such photoproteins was employed routinely in microbiology and was under investigation in the oil and gas industries, and work then began to develop the potential of photoproteins for detection of Ca2^+^ released from teeth during the demineralisation process of caries development.

A photoprotein is 'a naturally occurring bioluminescent protein capable of emitting light'.^20^ Most photoproteins used in biotechnology, such as aequorin and symplectin, originate from marine organisms including jellyfish, hydrozoa and squid. First isolated in the 1960s, these photoproteins emit a luminescent signal in the presence of free Ca2^+^, even at very low levels, due to an intramolecular reaction that conformationally changes the 'chromophore' site.^21^^,^^22^ Unlike fluorescent markers, photoproteins don't need an excitation source to produce luminescence.^22^ This is especially advantageous when looking at naturally fluorescent enamel as any stimulation of such could skew results.

## Development of a mechanism to assess lesion activity in enamel

The vision of Longbottom and Pitts was of a device that could help practice-based dentists manage caries better in their patients by reliably showing which lesions were active in a single visit, leading to targeted preventive care where needed, or reassurance that lesions were inactive where this was the case.

Focusing on the idea that photoproteins could be used to detect the presence of free Ca2^+^ and hence, active demineralisation, collaboration was sought within companies who were developing such technologies for the oil and gas industries. Edinburgh-based biotechnology company 'Lux Innovate Ltd' was identified and work began to develop the detection luminescence system and photoprotein.^23^^,^^24^^,^^25^^,^^26^ Initial patent applications were filed in 2007 and 2011.^27^^,^^28^

Early experiments were based on aequorin, which has a broad luminescence spectrum with a peak at around 460-470 nm.^20^ The imaging system to detect the photoprotein-induced Ca2^+^ signal involved the development of a low light charge-coupled device. Imaging mechanisms for detecting bioluminescence were developed along a similar timeline to the photoproteins. These predominantly work by converting the photoprotein's bioluminescent signal into electrons in a vacuum, whose signal is then amplified and output recorded on a phosphor screen as photons.^22^ Studies were carried out with Lux Innovate on extracted pre-molar teeth with lesions created in a specific site via acid application and on extracted teeth with white spot lesions. These proved the validity of the photoprotein/imaging device. Studies also showed that aequorin does not detect resin fillings.^27^

Spurred by the potential of the patents, Adam Christie (CEO) and Bruce Vernon (CTO) acquired them from Lux Innovate and the University of Dundee and set up the company Calcivis in Edinburgh in 2012, which dedicated itself to the development of what would become the Calcivis Imaging System. This work has been presented at a number of conferences;^23^^,^^24^^,^^25^^,^^26^^,^^29^^,^^30^^,^^31^ each step brought forth refinements. In addition, key aspects of the research work have been summarised recently by Longbottom and Vernon.^18^ Investment funding came from Archangels Investors in Edinburgh and the Scottish Investment Bank, together with EU Horizon 2020 and Scottish Enterprise grants. The first device received its medical device CE mark in December 2013, proving its alignment to regulatory standards in the EU.

Following the 2013 academic relocation of Pitts and Longbottom to King's College London Dental Institute (now Faculty of Dentistry, Oral and Craniofacial Sciences), collaboration continued, with the researchers (now through King's) setting up a caries activity study group at a European Organisation for Caries Research (ORCA) conference, which later developed into the Activity in Caries and Erosive Tooth Wear (ACE) Network.

## Method

### The Calcivis Imaging System

**Fig. 1:**
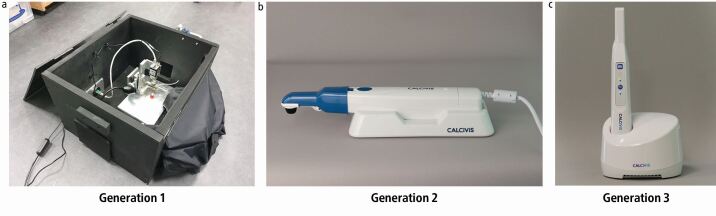
a, b, c) The three generations of the Calcivis System: Generation 2 was released in 2018, Generation 3 was released in September 2021

The Calcivis System (Fig. 1) has evolved over several generations. Initially, a light tight 'Black Box' was used to capture any light flash from the photoprotein onto a CCD image sensor. The Generation 2 device comprised an ergonomic handheld imaging sensor in a handpiece that also holds the photoprotein to be directed by the operator at the tooth surface under examination; the sensor is linked by cable to a laptop computer. The Generation 3 device is a further evolution in which the handpiece has been reduced in size, made more ergonomic and is now wirelessly connected to a host laptop computer.

**Fig. 2:**
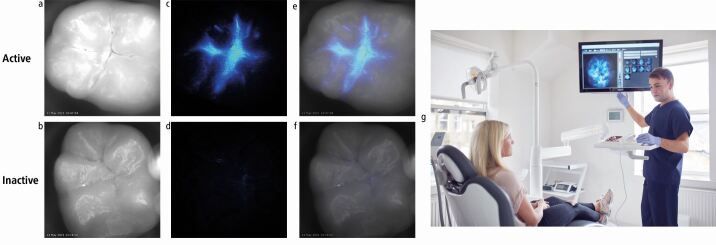
Imaging the Demineralisation Maps - active and inactive lesions. a, b) A direct photograph. c, d) Shows the presence or absence of a phosphoprotein signal of activity. e, f) A composite of the first two images of each series - which is displayed on the computer monitor. g) The in-surgery photograph is an example of how the Demineralisation Maps are used to communicate the results of the assessment to a patient. Images reproduced with permission from Calcivis

To operate the system, the handpiece tip is aimed at a clean and clinically air-dried tooth surface and the trigger operated. The device then automatically sprays 25 ml of the phosphoprotein directly onto the tooth surface and rapidly triggers the camera in order to capture any instantaneous light flash. In less than a second, a black and white image of the surface is taken; any flash of luminescence occurring where free Ca2^+^ is present is picked up by the device sensor. The result, presented on a computer screen by dedicated Calcivis software, is a composite picture of the black and white image and the luminescence signal. This allows visualisation of a 'demineralisation map' that can be interpreted by the dental professional and presented to the patient. As well as presence/absence of lesion activity, the hue of the photoprotein image on the spectrum of the 'royal blue' software indicates the level of ongoing calcium loss (Fig. 2). Figure 2 shows on the top row the sequence of three images for an 'active' lesion (clinical image, luminescence signal - if any - and composite of images one and 2), then the matching three images for an inactive lesion on the bottom row, with the images being discussed with a patient in the dental chair on the right side. An active lesion can be recognised by the dentist (and the patient when viewing the stored image) as the bright blue patch of light superimposed on the fissure pattern of the occlusal surface. An inactive lesion, however, appears as a straightforward, magnified image of an occlusal surface without any highlight at all.

Although the focus of this paper is on caries activity, it may be of interest to also be aware that the Calcivis Imaging System is also being developed as an aid to the clinical assessment and management of non-carious (erosive) tooth surface loss which is an increasingly common problem. This exciting parallel application is being developed with a range of laboratory experiments.

In order to understand what other technologies are currently available for evaluating carious lesion activity in clinical practice and the evidence around their performance, it is helpful to look at a (2018) international systematic review.^32^ This revealed four groups of available techniques: 1) systems based on combinations of visual and tactile criteria; and devices based on 2) pH assessment; 3) fluorescence; or 4) bioluminescence (the Calcivis System). The review highlighted that 'lesion activity is an important component to be taken into account when making decisions as to appropriate clinical caries management' and highlighted that, in busy practice settings, 'there is a crucial need for devices/tools that should, ideally, give instantly a straightforward "yes/no" answer (in the current context "active/inactive")'.^32^ Although the review predated the full publication of *in vivo* data from the Calcivis System, one of its two recommendations for caries activity assessment was 'to encourage *in vivo*…devices based on fluorescence and bioluminescence in both primary and permanent teeth'.^32^

Following development of the system, Calcivis senior scientist Mr Graham Bailey led further testing concentrated on examining specificity and sensitivity. Using extracted teeth bearing initial or moderate lesions, lesion extent and activity was first estimated and classified using the Nyvad^33^ and ICDAS^34^ scoring systems. In the first study,^35^ it was ascertained that specificity and sensitivity was greater with ICDAS. A subsequent *in vitro* study, utilising ICDAS and the Calcivis System, found that while the positive predictive value (detection of active lesions) was similar for both visual and Calcivis assessment methods, negative predictive value was higher with Calcivis.^36^ A recent overview of the initial experiments of this novel bioluminescence method for imaging demineralisation provides further details of the methodology and *in vitro* results.^37^

## Results

### Clinical validation

Testing of a version of the commercial device in a real-world setting for a US Federal Drug Administration-guided study began by recruiting several dental practices in Scotland. This was a pivotal part of the development of the Calcivis System as it was the first chance to assess not only how the system worked in dental practice, but how it fitted into a single dental visit, including what was needed regarding dental professional training and the patient's view of the process and results. The development and execution of the clinical study protocol was led by Marjory Willins, a highly experienced medical device clinical researcher at Calcivis, and the work included collaboration with five general dental practitioners headed up by Edinburgh-based Principal Investigator, Neil Shanks.

Results of the clinical study, which involved 110 participants aged 7-74 years, have also recently been published.^38^ The study demonstrated that with a high level of agreement, the Calcivis Imaging System can differentiate tooth surfaces clinically identified as involving active enamel lesions (ICDAS code 2/3) from sound sites (biochemically equivalent to inactive lesions) and that the system is safe for clinical use. This *in vivo* study showed that of the 90 teeth assessed as sound/equivalent to inactive lesions, 88 showed no bright light (bioluminescent signal) - a negative percentage agreement of 97.8%; while with the 86 teeth assessed as having active lesions, 78 showed a bright light (bioluminescent signal) - a positive percentage agreement of 90.7%.

The results also revealed a good degree of patient satisfaction. Participants rated their overall experience as good and over 96% found seeing the results helpful. Comments included: 'It was very quick and everything was explained clearly' and 'I now know exactly what areas to target to maintain my teeth'. Of the ten dental professionals (dentists and dental nurses) involved in the study, most found the system 'easy' or 'neither easy nor difficult' to prepare and use. The other smaller clinical studies carried out have supported the above findings regarding validity, ease of use and improvements in dentist-patient communications of the Calcivis System.

### The regulatory framework

The development of any new dental device technology requires the developer to pay close attention to the prevailing regulatory standards in those countries where the product will ultimately be supplied. Although Calcivis is based in Scotland, the company's short-term business aspirations are focused on the US. The combination of US market size, propensity to adopt new dental technology and the mixed private pay/insurance reimbursement ecosystem means that US launch is likely to make a return for the investors who funded the research and development more quickly than other markets. To this end, Calcivis has made regulatory filings to the US Food and Drug Administration in support of US market access. The unprecedented nature of the Calcivis device and the incorporation of a novel recombinant protein as an imaging agent meant that the Calcivis Imaging System is being held to the highest medical device standards and has been required to make a Premarket Authorisation (PMA) filing. While this has been a lengthy and involved process, the company anticipates final approval of the PMA this year, which would allow US product launch in 2022.

### Integration of the Calcivis System into dental practice

Monitoring of dental health is vital and it should be standard that assessments are charted at each visit for continuity of care.^39^^,^^40^ To this end, caries detection systems such as ICDAS (used to visually classify lesions) and the International Caries Classification and Management System (ICCMS), which extends ICDAS to encompass planning, management and review, have been developed to code each lesion so as to monitor its progress over time.^41^^,^^42^

The consensus of a 2012 workshop involving dental professionals, researchers, technology developers and insurers included that patients should be engaged 'with activities focused on understanding the caries disease process and creating caries preventive and behavioural norms at home' and that there is a need to 'develop and implement incentives to enhance the adoption of appropriate care of dental caries'.^40^ In 2016, the FDI World Dental Federation highlighted a number of challenges, including that there were a 'lack of tools (risk assessment tools, caries detection tools, caries activity assessment tools) and a lack of a systematic approach that can work in daily practice'.^43^

**Fig. 3:**
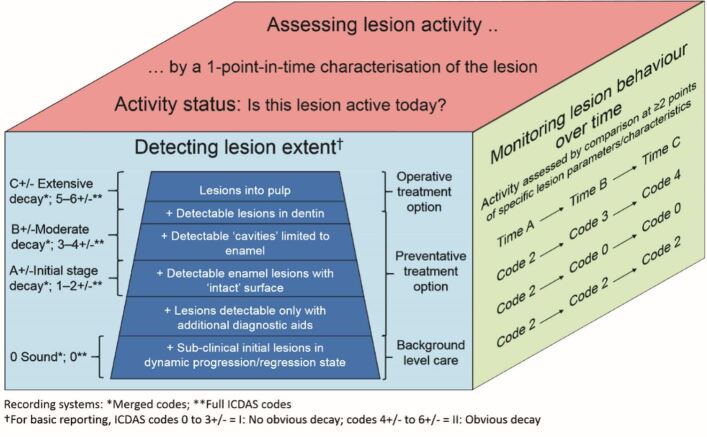
Linking lesion extent, monitoring and activity. This cube metaphor shows, on the front face, how lesion 'extent' is now staged clinically into the three categories of extensive, moderate or initial-stage decay according to ICDAS merged codes; the side face of the cube shows how lesions are traditionally monitored over time by comparison of serial clinical scores; while the top face of the cube shows the lesion activity domain - where an assessment of lesion activity at one time point is made

With these calls for improvement in mind, how does the Calcivis System fit into current caries detection, assessment and prevention protocol? According to the ICCMS, a personalised caries management plan needs to include patient history, including details of at-risk behaviours, a synthesis and diagnosis of individual lesions and overall caries risk, and a review of outcomes of any management decisions. The ICCMS incorporates a 'wardrobe' of assessment and management tools and options for the dental professional to choose from. The recent CariesCare Practice Guide^44^ provides a practice-friendly approach to its use. Coding of caries can range from simple (I: no obvious decay; II: obvious decay) to more detailed (ICDAS codes from 0-6±) or a merged system (from 0: sound to C±: extensive decay) (Fig. 3).^42^

The Calcivis System is currently anticipated to be used for ICDAS codes 1-4 lesions, as it can help identify and assess enamel lesion activity.

Market research^45^ has indicated that dental professionals will use the system to help communicate with their patients why an oral hygiene regimen, including dietary advice, is necessary and aid in decisions between preventive or interventional measures for each identified lesion site. The estimate of future caries risk should include analysis of lesion activity, which can be integrated into a caries 'likelihood' matrix to indicate low, moderate or high likelihood of new caries lesions developing.^42^ In all cases, the Calcivis System can then be used to monitor lesions over time to, hopefully, show the patient the results of any preventive measures applied.

A key advantage of the Calcivis System over indicators that highlight bacterial by-products or components is that, as well as being used for carious lesions, it can detect enamel demineralisation due to erosive tooth wear, a different condition involving non-carious demineralisation.^46^ This refined technology should help dentists improve delivery of risk-adjusted preventive clinical management of both caries and erosive tooth wear. Such management can be basic instructions to aid dental health, including oral hygiene measures, use of enhanced fluoride formulations and dietary advice, or in-clinic procedures, such as sealants, varnishes, gels and foams for at-risk surfaces.^40^^,^^42^

## Conclusion

The development of the Calcivis System was possible because of effective long-term collaborations between disparate groups of people, from dental and other academics, entrepreneurs, the business and investment community, general practitioners and their teams and patients. They all came to share the common vision that it would be possible to harness a new technology to help dental professionals provide better care for their patients' oral health.
